# Tyrosine kinase inhibitors and molecularly driven therapeutic approaches for canine lymphoma: a systematic review and meta-analysis

**DOI:** 10.3389/fvets.2026.1852091

**Published:** 2026-08-12

**Authors:** Yali Chen, Yu Gao, Shumin Li, Bo Liu, Ingo Nolte, Hugo Murua Escobar, Feng Yu

**Affiliations:** 1 Department of Small Animal Medicine, College of Veterinary Medicine, China Agricultural University, Beijing, China; 2 Department of Large Animal Medicine, College of Veterinary Medicine, China Agricultural University, Beijing, China; 3 Department of Small Animal Medicine and Surgery, University of Veterinary Medicine Hannover, Hannover, Germany; 4 Department of Clinical Veterinary Medicine, College of Veterinary Medicine, Jilin University, Changchun, Jilin, China; 5 Veterinary Teaching Hospital, China Agricultural University, Beijing, China; 6 Institute of Medical Genetics, University Medical Center Rostock, Rostock, Germany; 7 ECLN, European Canine Lymphoma Network, Rostock, Germany

**Keywords:** canine, clinical trials, lymphoma, meta-analysis, tyrosine kinase inhibitors

## Abstract

**Background:**

Canine lymphoma (cL), the second most common canine cancer after mammary carcinoma, presents therapeutic challenges due to limitations of conventional regimens, necessitating novel therapeutic approaches. Tyrosine kinase inhibitors (TKIs), which have demonstrated efficacy in human non-Hodgkin lymphoma (NHL), are increasingly being investigated as targeted therapies for cL, which also serves as a valuable translational model for NHL research.

**Objectives:**

To systematically evaluate the efficacy of TKIs in the treatment of cL.

**Methods:**

A comprehensive search of PubMed, Web of Science, Cochrane, and Embase databases was conducted, encompassing studies published between January 1, 2004 and January 1, 2026, using controlled vocabulary terms including “Canine,” “Dogs,” “Lymphoma,” and “Tyrosine Kinase Inhibitors”. A meta-analysis was subsequently performed to evaluate the efficacy of TKIs.

**Results:**

Seventeen articles were included, comprising 11 clinical trials and 9 *in vitro* experiments, with some studies encompassing both methodologies. A meta-analysis encompassing 101 dogs from 11 clinical trials showed an objective response rate (ORR) of 42% (95% CI: 32–53%; common-effect model), indicating moderate antitumor activity. Notably, canine T-cell lymphoma (ORR = 0.63) may exhibit a higher response rate compared to canine B-cell lymphoma (ORR = 0.27).

**Conclusions:**

TKIs show moderate therapeutic activity in cL and support the role of dogs as a translational model for NHL research. However, further studies are needed to confirm their efficacy and safety and to explore optimal combination and personalized treatment strategies.

## 1 Introduction

Canine lymphoma (cL) has emerged as a highly relevant model for studying human non-Hodgkin lymphoma (NHL) pathogenesis and treatment. Compared to rodents, cL may share a closer evolutionary relationship with humans (1), including a longer lifespan and more similar metabolic processes (2, 3), allowing for more accurate pharmacokinetic/pharmacodynamic (PK/PD) data. Epidemiologically, cL has an incidence rate of 13–114 cases per 100,000 dogs (4, 5) and is second only to mammary cancer as the most common cancer in canines (6). It is classified into B-cell (cBCL; 65–75% of cases) and T-cell (cTCL; 25–35% of cases) subtypes (7, 8) based on immunophenotypic characterization, paralleling human NHL, in which diffuse large B-cell lymphoma (DLBCL) predominates, while peripheral T-cell lymphoma (PTCL, approximately 6%) and cutaneous T-cell lymphoma (CTCL, approximately 4%) (9–11) account for a minority. In dogs, epidemiological data also indicate breed-specific predispositions, with Bullmastiffs, Rottweilers, and Scottish terriers exhibiting higher cL incidence (12–16).

At the molecular level, cL exhibits substantial overlap with NHL, including recurrent *MYC* amplification, *CDKN2A* mutations, oncogenic *FLT3* variants, and dysregulation of BCL-2 family proteins (17), as well as aberrant B-cell receptor (BCR) signaling and constitutive NF-κB activation (17, 18). Genomic analyses reveal striking molecular convergence between cL and NHL. Whole-genome sequencing studies demonstrate that 29/48 (60.4%) of common human cancer driver genes exhibit homologous mutations in cL, with tyrosine kinase (TKs) genes representing a significant proportion (13/29, 44.8%), suggesting cross-species conservation of oncogenic pathway activation mechanisms (19). Notably, tumor suppressor genes (TSGs) also display cross-species consistency, including frequent *TP53* mutations (20), and comparable inactivation rates of *TRAF3* (21, 22) and *FBXW7* (23) in cBCL and human DLBCL. While this molecular homology highlights the translational relevance of cL, current therapeutic strategies primarily target druggable proto-oncogenes such as TKs due to the challenges of restoring TSG function.

Pathway-level analyses identify 74 shared signaling pathways, including B-cell activation, JAK-STAT signaling cascade, lymphocyte differentiation, and immune responses (24). These pathways frequently involve TKs, exemplified by Bruton's tyrosine kinase (BTK). Notably, chromosomal instability of canine chromosome 13 (chr13) results in coordinated amplification of *MYC* (25), *PDGFR*α (25, 26), and *KIT* (25) further reinforcing TKs-driven oncogenic network (27). Collectively, these findings position TKs-related signaling as a central and druggable axis in cL.

Despite these molecular insights, current therapies for cL remain sub-optimal. CHOP-based chemotherapy (CHOP: cyclophosphamide, doxorubicin, vincristine, and prednisone) achieves remission rates of 80–90%, but median survival time is limited to 12–16 months, with only 20–25% surviving for 2 years (28) and the vast majority of the dogs with multicentric lymphoma eventually relapsed (28). Outcomes are poorer in cTCL than cBCL (29, 30). In human lymphoma, after first-line treatment with R-CHOP (R: rituximab), 40% of patients with DLBCL (the most prevalent BCL subtype) experience relapsed or refractory (R/R) disease (31), with worse outcomes observed in PTCL (32). These limitations highlight a shared unmet clinical need across species.

Accordingly, this study systematically evaluates *in vitro* and *in vivo* evidence from January 1, 2004 to January 1, 2026 through systematic review and meta-analysis to assess the therapeutic potential of tyrosine kinase inhibitors (TKIs) in cL, with the aim of informing both veterinary and translational oncology.

## 2 Materials and methods

This systematic review and meta-analysis followed the Preferred Reporting Items for Systematic Reviews and Meta-Analysis (PRISMA) guidelines (33).

### 2.1 Inclusion and exclusion criteria

Study inclusion and exclusion criteria using the PICOTS framework, evaluated tyrosine kinase inhibitors (TKIs) as monotherapy or combined with other agents in canine spontaneous lymphoma or *in vitro* lymphoma cell line models, encompassing prospective/retrospective clinical trials, cohort studies, or experimental designs without control group restrictions; primary outcomes included objective response rate (ORR), RECIST-defined responses (complete response[CR] or partial response[PR]), and *in vitro* IC_50_/dose-response profiles, excluding duplicate publications, non-English records, non-eligible publication types (reviews, conference proceedings, meeting records, editorial materials, case reports and other non-eligible articles), TKIs-unrelated interventions, non-compliant outcomes, or non-conforming study designs. Conference proceedings and meeting records were excluded; however, conference abstracts were not automatically excluded from this review. Instead, they were assessed using the same eligibility criteria as full-text publications and were included when sufficient methodological information and outcome data were available for data extraction and analysis. The detailed content is shown in Table 1.

**Table 1:** Inclusion and exclusion criteria.

*PICOTS*	*Inclusion criteria*	*Exclusion criteria*
**Population (P)**	Canine subjects with spontaneous lymphoma or *in vitro* studies utilizing canine lymphoma cell lines.	Canine subjects with other diseases.
**Interventions (I)**	Administration of tyrosine kinase inhibitors (TKIs), either as monotherapy or in combination with other agents.	Interventions not involving TKIs or TKIs-based therapies.
**Control (C)**	No restriction based on whether the study had a control group.	No restriction based on whether the study had a control group.
**Outcome (O)**	(1) Primary outcomes included objective response rate (ORR) and treatment response, defined by RECIST criteria (complete response [CR], partial response [PR]). (2) For *in vitro* studies, IC_50_ and dose-response profiles were evaluated.	(1) Non-quantifiable clinical endpoints (e.g., descriptive or qualitative response data). (2) Non-standardized scoring systems (e.g., in-house tumor grading not based on RECIST criteria). (3) *In vitro* studies lacking IC_50_ values or dose–response data. (4) Biomarker-only studies without direct correlation to treatment efficacy.
**Timeframe (T)**	Published between January 1, 2004, and October 26, 2025	(1) Studies published outside the specified date range. (2) Preprints not yet peer-reviewed by October 26, 2025.
**Study Design (S)**	Prospective study or retrospective study, including clinical trials, cohort studies, or experimental studies.	Studies not involving original clinical or experimental data, such as reviews, case reports, editorials, and conference abstracts.
Other Exclusion Criteria	/	(1) Duplicate publications. (2) Non-English language articles. (3) Records with unavailable full text or insufficient outcome data. (4) Retracted or database-flagged ineligible articles. (5) Studies involving animals without an explicit statement of ethical approval

### 2.2 Data sources and searches

Firstly, the study searched PubMed, Web of Science, Cochrane, and Embase databases, with the publication year set to 2004.01.01–2026.01.01. The search strategy was constructed using both a controlled vocabulary (MeSH in PubMed and Cochrane, EMTREE in Embase, and direct keywords in Web of Science) and a wide range of free-text terms. All retrieval strategies were determined after multiple preliminary retrievals. The controlled terms included “Canine,” “Dogs,” “Lymphoma,” and “Tyrosine Kinase Inhibitors”. Publications were limited to those written in English. The detailed PubMed search strategy is provided in Supplementary Material, and equivalent search strategies adapted to each database were applied for Embase, Web of Science, and Cochrane Library.

### 2.3 Literature screening and data extraction

Two review authors selected the papers and screened the literature stringently according to the predetermined inclusion and exclusion criteria, and the selections were cross-checked. They resolved the disagreement by consensus. Firstly, researchers read the abstracts and titles to exclude articles that did not meet the criteria, and then read the full texts of the remaining articles to finally include eligible articles. Data extracted from eligible articles included: (1) basic information of the article, such as authors, publication year, language, and type of article; (2) information about the dogs, such as the type of lymphoma, the name, source, and mutated genes of the canine lymphoma cell lines; (3) TKIs agent, dosage, combination therapies, molecular targets; (4) outcome, i.e., PR, CR, and ORR; and the response of cell lines to drugs and IC_50_. When multiple publications shared overlapping patient datasets, only the most complete report was retained for data extraction.

### 2.4 Methodological quality assessment

The methodological index for non-randomized studies (MINORS) was used to evaluate the methodological quality of the included studies. The following items were evaluated (34): (1) a clearly stated objective (the question addressed should be precise and supported by solid evidence from literature), (2) inclusion of consecutive patients (all patients potentially fit for inclusion have been included in the study during the study period with no exclusion or details about the reasons for exclusion), (3) prospective collection of data (data was collected according to the protocol established before the beginning of research), (4) endpoints appropriateness (unambiguous explanation of the criteria used to evaluate the main outcome), (5) unbiased assessment of the study endpoint (blind evaluation of objective endpoints and double-blind evaluation of subjective endpoints), (6) follow-up period appropriate to the aim of the study (the follow-up period should be sufficiently long), (7) loss to follow-up less than 5% (try to bring into all patients in the follow-up as far as possible), and (8) prospective calculation of the study size (the sample size estimation should account for expected incidence of the outcome event and corresponding 95% CI, information about the level for statistical significance and estimates of power when comparing the outcomes). Each item was scored on a scale of 0–2, where 0 indicated unreported, 1 indicated underreported, and 2 indicated adequately reported. Methodological quality was independently assessed by two reviewers using the MINORS tool, and disagreements were resolved by consensus.

### 2.5 Statistical analysis

A meta-analysis of the ORR was conducted using R 4.5.1. Heterogeneity among studies was estimated using the chi-square test and the *I*^2^ statistic. If the *P*-value was less than 0.05 and/or the *I*^2^ value was greater than 50%, it was considered that there was substantial heterogeneity among the included studies, and a random-effects model was used for the combined analysis. Otherwise, a common-effect model was applied. Under special circumstances, considering the limited sample size and potential uncertainty in heterogeneity estimation, both common-effect and random-effects models were applied, and their results were reported to enhance the robustness and transparency of the analysis. Additionally, GraphPad Prism 10 was utilized to assist in the creation of graphical representations.

## 3 Results

### 3.1 Literature search

**Figure 1:**
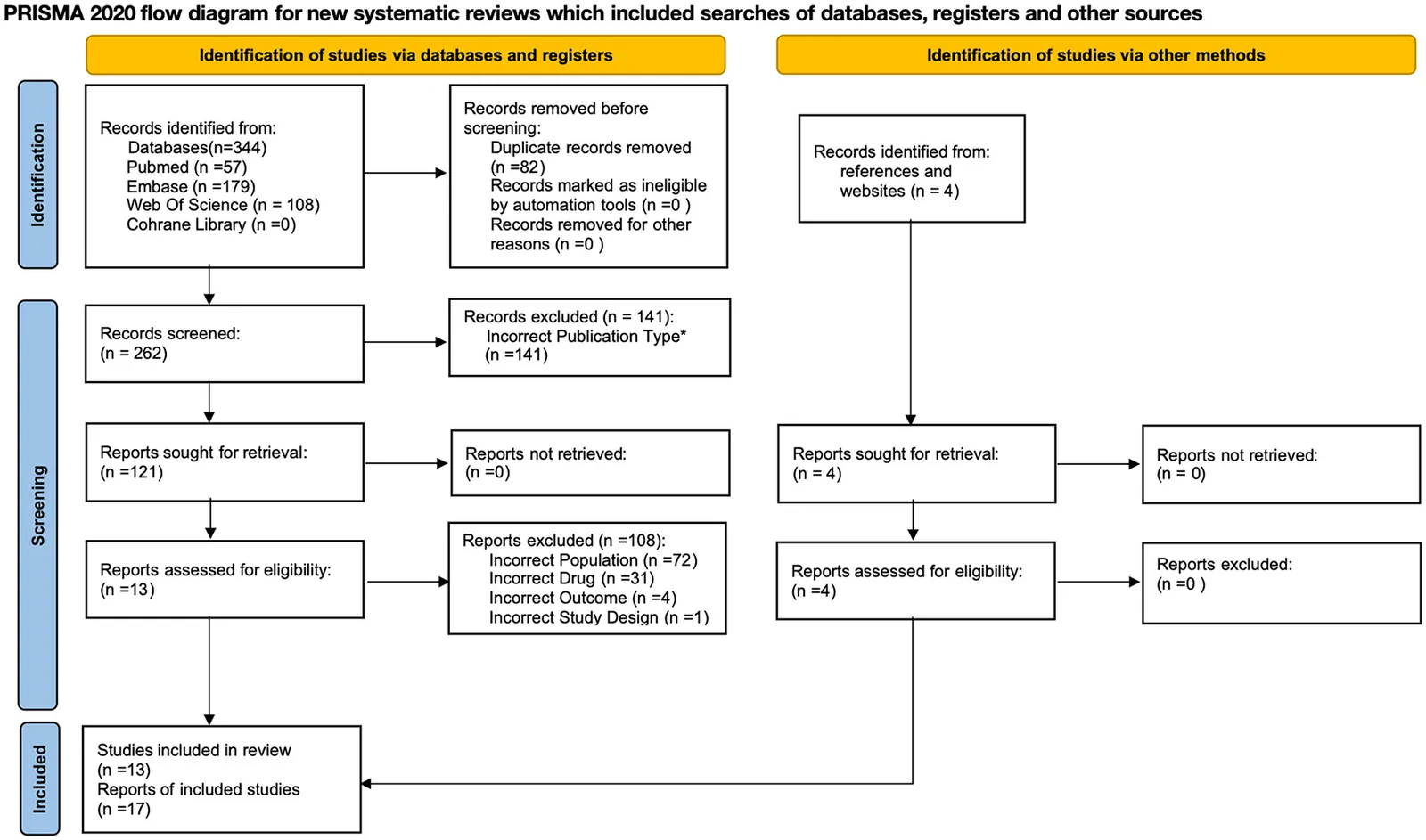
PRISMA flow diagram of the study selection process. *According to the language type (not in English), article type (reviews, conference proceedings, meeting records, editorial materials, case reports and other non-eligible articles).

A total of 344 articles were identified through the four databases. After removing 82 duplicate articles, 262 articles remained to be screened. Based on a preliminary screening of titles and abstracts, 141 non-conforming publications were excluded, resulting in 121 articles. Subsequently, based on the PICOTS criteria, articles were excluded for the following reasons: incorrect population (*n* = 72, e.g. studies not focusing on cL), incorrect drug interventions (*n* = 31, e.g., studies not involving TKIs), outcomes not meeting inclusion criteria (*n* = 4, e.g., studies reporting descriptive or qualitative response data only), and incorrect experimental design (*n* = 1, e.g., the study focused on different cell lines rather than assessing TKIs-based interventions). This left 13 eligible articles from the database searches. Additionally, 4 eligible articles were identified through references and websites. The process of article retrieval is presented in Figure 1.

### 3.2 Basic information of included studies

As one publication reported on two distinct clinical trial groups, these were treated as separate studies in our analysis. Consequently, the 17 articles yielded data for 11 clinical trials and 9 *in vitro* experiments. Ten clinical trials were prospective studies while another was a retrospective study (35). All studies were single-arm clinical trials. A total of 101 dogs with the condition were included, with the sample size per study ranging from 3 to 20. Four trials exclusively focused on BCL (36–39) whereas four trials con-centrated specifically on TCL (40–42). Yamazaki et al. (43) included both BCL and TCL dogs, while Pan et al. (44) did not perform any immunophenotyping. Additionally, the study of Baba et al. (45) included eight dogs, only two of which were confirmed as TCL. The clinical trials evaluated TKIs targeting several distinct signaling pathways. In BCL, the investigated agents primarily included BTK inhibitors (PCI-32765, ACP-196, acalabrutinib, and SY-1530) and the multi-target inhibitor toceranib, which targets KIT, VEGFR, and PDGFR. In TCL, the evaluated agents mainly consisted of masitinib (c-KIT and PDGFR inhibitor), CPI-818 (ITK inhibitor), oclacitinib (JAK family inhibitor), and toceranib. Collectively, these agents targeted both receptor tyrosine kinases (KIT, PDGFR, and VEGFR) and intracellular non-receptor tyrosine kinases, including BTK, ITK, and members of the JAK family. The canine lymphoma cell lines used in the 9 *in vitro e*xperiments included CLBL-1 (4 studies) (38, 46–48), GL-40 (2 studies) (49, 50), CLBL1–8.0 (1 study) (51), CLGL-90 (1 study) (52), OSW (1 study) (40), and 17–71 (1 study) (52). In addition, GL-1 (4 studies) was used as the control cell line (47, 49, 50, 52). The information on clinical trials and cell lines has been listed in Tables 2, 3.

**Table 2:** The efficacy of different TKIs in clinical trials.

References	Diseases	Drug	Events (CR+OR)	*n*	Proportion (ORR)	95% CI	Weight%
**BCL Subgroup**			**14**	**53**	**0.27**	**[0.17; 0.41]**	**51.9**
Honigberg et al. (36)	B cell lymphoma	PCI−32765 (BTK inhibitors)	3	8	0.38	[0.09; 0.76]	9.4
Gardner et al. (37)	B cell non–Hodgkin lymphoma	ACP−196 (BTK inhibitors)	3	12	0.25	[0.05; 0.57]	11.2
Harrington et al. (38)	B cell non–Hodgkin lymphoma	Acalabrutinib (BTK inhibitors)	5	20	0.25	[0.09; 0.49]	18.7
Yamazaki et al. (43)	Multidrug–resistant (MDR)canine B cell lymphoma	Toceranib (Palladia) (PDGFR, VEGFR, and KIT inhibitors)	0	3	0.00	[0.00; 0.71]	2.2
Wang et al. (39)	B cell lymphoma	SY−1530(BTK inhibitors)	3	10	0.30	[0.07; 0.65]	10.5
**Not Evaluated**			**2**	**9**	**0.34**	**[0.08; 0.77]**	**5.6**
Pan et al. (44)	Multicentric lymphoma	CCNU and toceranib (2.75 mg kg^−1^ PO EOD) therapy	2	3	0.67	[0.09; 0.99]	3.3
Baba et al. (45)	Canine epitheliotropic lymphoma	Oclacitinib (JAK1, JAK2, JAK3, tyrosine kinase 2 inhibitors)	0	6	0.00	[0.00; 0.46]	2.3
**TCL Subgroup**			**25**	**39**	**0.63**	**[0.47; 0.77]**	**42.7**
Hermine et al. (40)	Peripheral T cell Lymphoma	Masitinib (c–KIT and PDGFR inhibitors)	8	11	0.73	[0.39; 0.94]	10.8
Hermine et al. (40)	Peripheral T cell Lymphoma	Masitinib (c–KIT and PDGFR inhibitors)	5	11	0.45	[0.17; 0.77]	13.5
Holtermann et al. (41)	Canine epitheliotropic T cell lymphoma	Masitinib (c–KIT and PDGFR inhibitors)	7	10	0.70	[0.35; 0.93]	10.4
Yamazaki et al. (43)	Multidrug–resistant (MDR) T cell canine lymphoma	Toceranib (Palladia) (PDGFR, VEGFR, and KIT inhibitors)	2	2	1.00	[0.16; 1.00]	2.1
Thamm et al. (42)	T cell Lymphoma	CPI−818 (ITK inhibitors)	2	3	0.67	[0.09; 0.99]	3.3
Baba et al. (45)	Canine T cell epitheliotropic lymphoma	Oclacitinib (JAK1, JAK2, JAK3, tyrosine kinase 2 inhibitors)	1	2	0.50	[0.01; 0.99]	2.5
**Overall**			**41**	**101**	**0.42**	**[0.32; 0.53]**	**100**

Bold values indicate pooled estimates derived from the subgroup analyses (BCL, TCL, and not-evaluated groups) and the overall meta-analysis.

**Table 3:** The efficacy of different TKIs *in vitro* experiments.

Cell Line	Origin	Mutation	Drug	Response rate	Year, (Ref.)
GL-1	B-cell leukemia	FLT ITD (Internal Tandem Duplication)	Lestaurtinib (FLT3 inhibitors)	A dose-dependent growth inhibition of the GL-1 cells was evident, with an approximately 40% growth reduction after being exposed to 10 nM lestaurtinib for 96 hrs.	2011,
17-71	B-cell lymphoma	No FLT ITD		The 17-71 cell line was essentially resistant to the growth inhibitory effects of lestaurtinib.	
CLGL-90	Chronic large granular T cell leukemia	No FLT ITD		The CLGL-90 cell line showed only a modest reduction in growth.	
GL-1	B-cell leukemia	Not mentioned	Masitinib (c-Kit and PDGFR inhibitors), combination of masitinib and doxorubicin	1. Masitinib decreased cell proliferation equally in both GL-1 and GL-40 cells. 2. The inhibitors effect appeared concentration dependent, and a more than 80% reduction (compared with untreated controls) was observed after a 24 h incubation with masitinib at a concentration of 40 μm. 3. After 48 h of incubation with 10 μm masitinib, cell proliferation decreased to 83 ± 6% and 78 ± 7% of the controls for, respectively, the GL-1 and GL-40 cell lines. 4. Although masitinib at concentrations of 1 and 10 μm had no additional effect on doxorubicin-induced cytotoxicity in the GL-1 cells, it enhanced the antiproliferative effect of doxorubicin on GL-40 cells as demonstrated by a significant decrease in the IC_50_ value, from 48ng/ml to 15ng/ml.	2013, (48)
GL-40	A doxorubicin / vincristine resistant subline created from GL-1, a canine B-cell lymphoma cell line with P-gp overexpression	Not mentioned			
OSW	Malignant pleural effusion of CPTCL	Not mentioned	Masitinib (c-Kit and PDGFR inhibitors)	Inhibition of cell proliferation was observed following single-agent masitinib treatment in the OSW canine T-cell lymphoma line, with an IC_50_ of 0.005 μM.	2015, (39)
CLBL-1	Affected peripheral lymph nodes	Not mentioned	Acalabrutinib (BTK inhibitors)	BCR signaling activates BTK and downstream targets in canine lymphoma cells, and inhibition of this signaling decreases the proliferation and survival of CLBL1 cells.	2016, (37)
GL-1	B-cell leukemia	Not mentioned	BI-2536 (VEGFR and PDGFR inhibitors), Sorafenib (VEGFR, PDGFR and Raf inhibitors), Quizartinib (FLT3 inhibitors), Sunitinib (VEGFR, PDGFR and KIT inhibitors), Toceranib (KIT, VEGFR2 and PDGFR2 inhibitors).	IC_50_: < 0.1 μM	2018, (49)
			TAE684 (ALK inhibitors), SGI-1776 (Pim inhibitors), TAE226 (FAK inhibitors), Tozasertib (pan-Aurora inhibitors).	IC_50_: 0.1–1 μM	
			Dasatinib (Abelson tyrosine kinase inhibitors), Pazopanib (VEGFR and FGFR inhibitors), Nilotinib (Abelson tyrosine kinase inhibitors), Erlotinib (EGFR inhibitors), Axitinib (VEGFR inhibitors).	IC_50_: 1–10 μM	
			Gefitinib (EGFR inhibitors), Imatinib (Abl inhibitors), Masitinib (c-Kit and PDGFR inhibitors), Lapatinib (EGFR and HER2 inhibitors), Vandetanib (EGFR, VEGFR and RET inhibitors).	IC_50_: >10 μM	
GL-40	A doxorubicin / vincristine resistant subline created from GL-1, a canine B-cell lymphoma cell line with P-gp overexpression	Not mentioned	The same applies above.	No relevant differences were found in IC_50_ between GL-1 and GL-40 for any of the PKIs tested.	
CLBL1-8.0	CLBL-1	Not mentioned	Imatinib (Abl inhibitors) and doxorubicin	1. The CLBL-1 cell line demonstrated high sensitivity to both doxorubicin and imatinib. The IC_50_ values for doxorubicin and imatinib in CLBL-1 cells were 0.7 ± 0.04 mM and 8.1 ± 0.43 mM, respectively. 2. The CLBL1-8.0 cells exhibited a 9.7-fold increase in resistance to doxorubicin compared to the parental CLBL-1 cells. The sensitivity to Imatinib remained statistically unchanged.	2019, (50)
CLBL-1	A male Bernese Mountain dog, which was diagnosed with DLBCL (stage IV).	Not mentioned	1. Entospletinib (0.001–10 μM; SYK inhibitors) 2. The combination of Entospletinib with I-BET151 (BET inhibitors) or AZD5153 (BET inhibitors)	1. Entospletinib (0.001–10 μM) in the mono-application did not show antiproliferative effect. 2. The combination of Entospletinib with I-BET151 or AZD5153 does not significantly enhance inhibition of proliferation and induction of apoptosis / necrosis in CLBL-1 cells.	2020, (45)
CLBL-1, GL-1	1. The CLBL-1 cell line was isolated from a male Bernese Mountain dog with confirmed DLBCL (stage IV). The GL-1 was derived from a German Shepherd.	*BTK, PI3K*	1. Ibrutinib (BTK inhibitors) 2. Combined Application of Ibrutinib and AS-605240 (phosphoinositide 3-kinase (PI3K) inhibitors)	1. Compared to the DMSO control, ibrutinib showed anti-proliferative effects on CLBL-1 beginning at 1 μM at all time points, while the reference cell lines GL-1 showed significant reduction at 2.5 μM at selective time points. 2. Compared to the control, 5 μM ibrutinib significantly increased CLBL-1 apoptosis/necrosis from 19.3% up to 81.5%. GL-1 showed a corresponding increase from 27.6% to 67.7%. 3. Compared to single treatments with ibrutinib (1 μM) or AS-605240 (2.5, 5, 10μM), combined application of Ibrutinib and AS-605240 enhances inhibition of CLBL-1 proliferation and metabolic activity and induces apoptosis and necrosis of CLBL-1.	2021, (46)
CLBL-1	The CLBL-1 cell line was isolated from a male Bernese Mountain dog with confirmed DLBCL (stage IV).	Not mentioned	Ibrutinib (BTK inhibitors) and AS-605240 (phosphoinositide 3-kinase (PI3K) inhibitors)	Based on the provided results, the combined treatment with 1 μM Ibrutinib and 5 μM AS-605240 significantly reduced the number of viable CLBL-1 cells to 2.7 million and decreased metabolic activity to 53.8%, demonstrating a synergistic anti-proliferative and anti-metabolic effect.	2025, (47)

CPTCL, Canine peripheral T cell lymphoma; CLBL-1, a canine B-cell lymphoma line; GL-1, a canine B-cell lymphoid cell line; GL-40, doxorubicin/vincristine resistant subline, a canine B-cell lymphoma cell line with P-gp overexpression; CLBL1-8.0 cell line, doxorubicin-resistant and established by serially increasing the concentration of doxorubicin in the parental CLBL-1 cell line during culture, up to a final concentration of 8 μm for approximately 8 months; BI-2536, inhibitor of Plk1 enzyme activity; TAE684, ALK inhibitor; SGI-1776, Pim inhibitor (member of Ser/Thr Protease inhibitor); TAE226, a dual inhibitor of FAK and insulin-like growth factor-I receptor (IGF-IR); DLBCL, diffuse large B-cell lymphoma.

### 3.3 Methodological quality assessment

Quality assessments were conducted for all clinical trials. All clinical trials listed a clearly stated aim, prospectively collected data, collected key endpoints, and had a loss to follow-up rate of less than 5%. Six clinical trials included consecutive patients, and ten clinical trials had an appropriate follow-up period for the aim of the study. None of the trials used blinding for the assessment of outcomes, and all trials failed to detail a prospective calculation of sample size. Ultimately, seven studies scored 13 points, and four studies scored 12 points. The results are shown in Table 4.

**Table 4:** The quality assessment results (0, high risk; 1, medium risk; 2, low risk).

References	A clearly stated objective	Inclusion of consecutive patients	Prospective collection of data	Endpoints collection of data	Unbiased assessment of the study endpoint	Follow–up period appropriateness	Loss to follow up less than 5%	Prospective calculation of the study size	Total
Honigberg et al. (36)	2	2	2	2	0	1	2	1	12
Gardner et al. (37)	2	1	2	2	0	2	2	1	12
Pan et al. (44)	2	2	2	2	0	2	2	1	13
Hermine et al. (40)	2	1	2	2	0	2	2	1	13
Hermine et al. (40)	2	1	2	2	0	2	2	1	13
Harrington et al. (38)	2	2	2	2	0	2	2	1	13
Holtermann et al. (41)	2	1	2	2	0	2	2	1	12
Yamazaki et al. (43)	2	2	2	2	0	2	2	1	13
Thamm et al. (42)	2	2	2	2	0	2	2	1	13
Wang et al. (39)	2	1	2	2	0	2	2	1	12
Baba et al. (45)	2	2	2	2	0	2	2	1	13

### 3.4 Meta-analysis results

#### 3.4.1 Clinical trials

**Figure 2:**
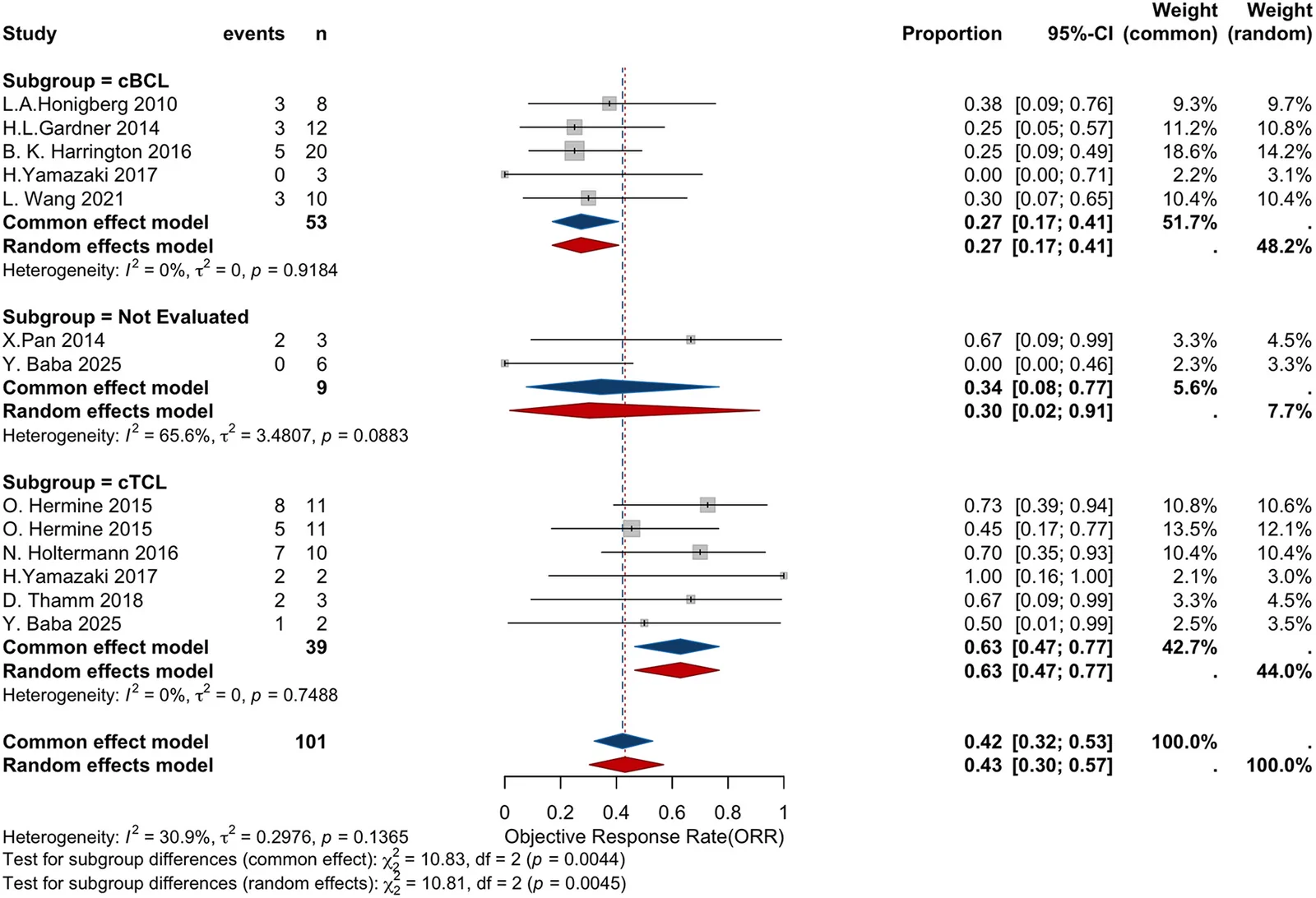
Forest plot showing the results of the meta-analysis of overall response rate (ORR) in cL. The plotted proportion represents the ORR, defined as the number of dogs achieving complete or partial response (CR+PR) divided by the total in each study (*n*). Pooled analysis shows a significantly higher ORR in cTCL (0.63) than in cBCL (0.27), *P*-value for subgroup difference less than 0.05. The “Not Evaluated” subgroup exhibited substantial heterogeneity (*I*^2^ = 65.6%), and its result is unreliable. Squares and diamonds represent individual and pooled estimates, respectively, with 95% CI.

A total of 11 clinical trials were included and subjected to a meta-analysis. Although one patient in the study by Wang et al. was reported as partial response/stable disease (PR/SD), we adopted a conservative approach in our meta-analysis by excluding this equivocal case from the PR category to maintain analytical rigor. Prompted by the presence of heterogeneity, subgroup analyses were conducted. After accounting for variables such as stages, substages, prior treatment status, combination therapies, and histological subtypes, the subgroup analysis revealed different treatment responses only across immunophenotypes (cBCL: 0.27 *vs*. cTCL: 0.63 *vs*. Not Evaluated: 0.34; *P* < 0.05). The “Not Evaluated” subgroup was defined as dogs that did not undergo immunophenotyping or were thus classified as neither T-cell nor B-cell origin. Further subgroup-specific details are provided in Table 2. The overall response rate (ORR) and 95% confidence interval (95% CI) for each study were analyzed based on the primary outcome measure of ORR. A total of 31 partial responses (PR) and 10 complete responses (CR) were observed. The overall effect size analyzed by the common-effect model was 0.42, with a 95% CI of (0.32–0.53). The weight of each study ranged from 2.1% to 18.6%, and the effect size varied between 0.00 and 1.00. However, the intervention (TKIs) showed a consistent trend in improving outcomes, indicating that TKIs have a certain efficacy in the treatment of cL. The figure of the forest plot is shown in Figure 2. Additionally, to evaluate the robustness of the results, a random-effects model was also employed for analysis, which produced a comparable pooled estimate (ORR = 0.43; 95% CI: 0.30–0.57) and is presented in the forest plot. Other subgroup analyses were shown in Supplementary Figures S1–S5.

#### 3.4.2 *In vitro* experiments

Among the 9 *in vitro* experiments included, only one study used a T-cell line, and the targets of the drugs used in the experiments were distributed across receptor tyrosine kinases, cytoplasmic tyrosine kinases, and nuclear tyrosine kinases. In the 9 *in vitro* experiments included, only 1 study (Entospletinib at 0.001–10μM) was considered to have no significant effect on CLBL-1 cell line (46). Five studies involved combination therapy; two of these combined TKIs with doxorubicin, and both demonstrated enhanced anti-proliferative effects against doxorubicin-resistant cell lines (49, 51). Another study that combined Entospletinib (SYK inhibitors) with BET inhibitors suggested that combination therapy did not show higher anti-proliferative effects (46). The remaining two studies that combined another type of TKIs (Ibrutinib, BTK inhibitors) with PI3K inhibitors exhibited a synergistic anti-proliferative and pro-apoptotic effect in the CLBL-1 cell line (47, 48), significantly enhancing growth inhibition compared with either agent alone. All information is shown in Table 3.

### 3.5 Publication bias and heterogeneity of included studies

**Figure 3:**
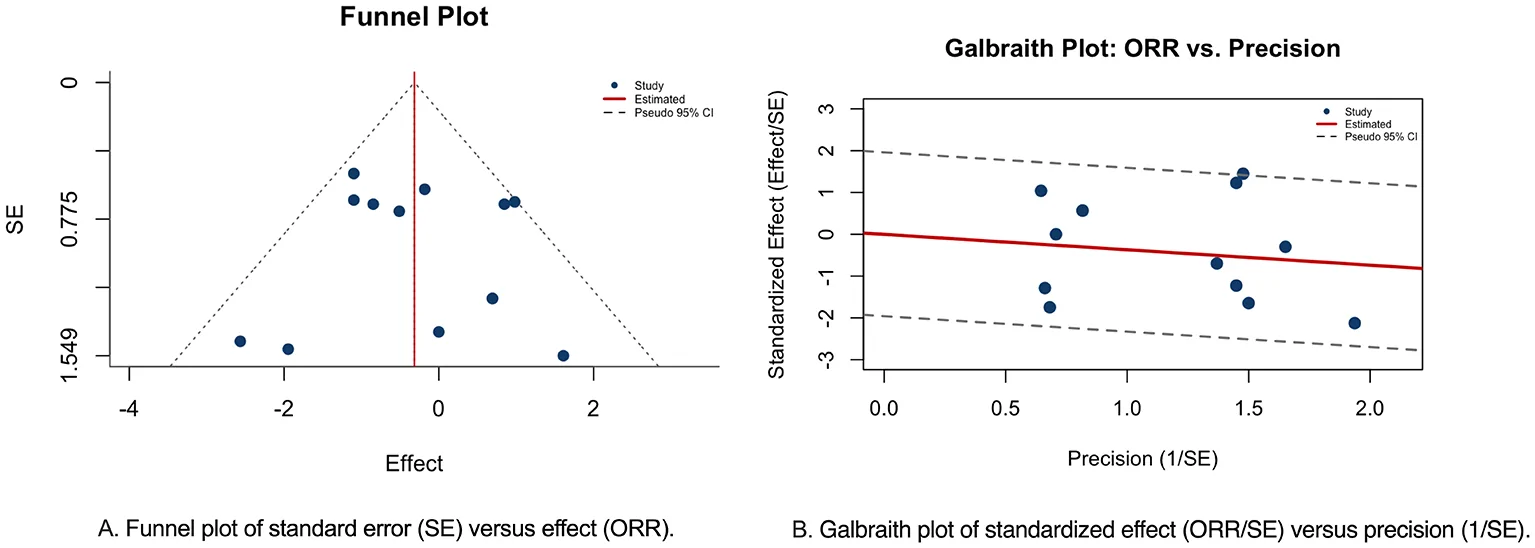
Funnel and Galbraith plots for assessing publication bias and heterogeneity in the meta-analysis of ORR. **(A)** Funnel plot, depicting the standard error (SE) of each study against its effect size (ORR). In the absence of significant publication bias, studies should distribute symmetrically in an inverted funnel shape around the pooled effect estimate. **(B)** Galbraith plot, displaying the standardized effect (Effect/SE) against precision (1/SE). This plot is used to visually identify outliers and assess heterogeneity. Studies that deviate substantially from the regression line through the origin are considered contributors to heterogeneity.

The test for publication bias revealed that the funnel plot (Figure 3A) was essentially symmetrical. Egger's test yielded a *P*-value greater than 0.05 (*P* = 0.4862). Therefore, we found no strong evidence of publication bias. A sensitivity analysis using a random-effects model confirmed the robustness of the primary results (ORR = 0.43; 95% CI: 0.30–0.57). Given that these tests may not be sensitive to studies with small sample sizes, the possibility of publication bias cannot be completely ruled out.

The study mainly used a common-effect model to combine effect sizes. The test for heterogeneity showed an *I*^2^ of 30.9% and a *P*-value of 0.1365. In cTCL and cBCL subgroups, *I*^2^ = 0 and *P*-values were greater than 0.05, indicating little heterogeneity within each which suggests that different immunophenotypes are likely the major source of overall heterogeneity observed in the entire research. In the Galbraith plot (Figure 3B), most of the points fell within the 95% confidence interval, indicating that the heterogeneity between studies was not significant.

## 4 Discussion

This paper synthesizes evidence from published clinical trials and *in vitro* experiments investigating cL management. Meta-analysis of pooled clinical data indicates that TKIs demonstrated moderate efficacy (ORR = 42%; 95% CI: 32%, 53%; common-effect model) in dogs with cL, but the small number of studies included in this meta-analysis necessitates cautious interpretation of these findings.

Although CHOP-based chemotherapy protocols achieve high initial remission rates in dogs with lymphoma, their long-term efficacy remains limited. A retrospective study involving 95 dogs with multicentric lymphoma that relapsed after a first-line 6-month CHOP protocol found that 78% responded to CHOP reinduction, but the median second remission duration (159 days) was shorter than the first (289 days). This progressive reduction in remission intervals indicates the development of drug resistance, underscoring a major limitation of CHOP-based therapy (53). Furthermore, immunophenotype is a key prognostic factor determining survival time and relapse in dogs with lymphoma. The risks of relapse and early death were significantly greater in cTCL than in cBCL, as evidenced by shorter remission (52 *vs*. 160 days, *P* < 0.001) and survival times (153 *vs*. 330 days, *P* < 0.001) (54). Given the poor prognosis of cTCL and the existence of relapse and resistance, novel therapeutic approaches are urgently needed.

In this presented analysis, although the Not Evaluated subgroup was included in the analysis, the interpretation of its results requires caution due to the limited number of studies and substantial heterogeneity (*I*^2^ = 65.6%), and thus should not be overinterpreted. Therefore, we primarily focus on the comparison between cBCL and cTCL, which demonstrate high internal consistency. Notably, cTCL (including 23 PTCL and 12 CTCL cases; ORR = 0.63) exhibited a markedly higher response rate compared with cBCL (ORR = 0.27). BTK inhibitors were predominantly investigated in BCL because BTK is a key mediator of B-cell receptor signaling and plays an important role in B-cell survival and proliferation. Conversely, ITK and JAK inhibitors were mainly evaluated in TCL, reflecting the importance of T-cell receptor signaling and JAK/STAT pathway activation in T-cell lymphomagenesis. Among these studies focused on cTCL, TKIs (Masitinib and Toceranib) mainly target PDGFR and KIT (40, 41, 43). However, direct and powerful evidence that can demonstrate corresponding upregulation of expression in included cTCL patients is lacking. The study by Hermine et al. (40) using masitinib was based on the premise of upregulated PDGFR expression in human TCL and lacked validation in canine models. In a complementary study, Holtermann et al. (41) using masitinib reported that KIT was negative in all 168 cTCL samples tested, while the variable positivity for PDGFR might be attributed to different mutation sites, potentially explaining the inconsistent drug (masitinib) efficacy among PDGFR-positive individuals. Moreover, Yamazaki et al. (43) using toceranib (PDGFR, VEGFR, and KIT inhibitors) found that PDGFR-α expression was higher in multidrug resistant cTCL dogs compared to treatment-naive dogs; although c-KIT did not differ between these cTCL groups, it was uniformly elevated relative to dogs with cBCL. Critically, this latter finding suggests that PDGFR upregulation may represent an adaptive mechanism to prior therapy rather than the initial driver of TKIs sensitivity. Thus, the clinical relevance of receptor tyrosine kinase (PDGFR and KIT) as primary therapeutic targets in cTCL remains uncertain. It is also important to note that masitinib targets other signaling pathways, including Lck, Lyn, FGFR3, and FAK (55). This broader targeting profile suggests that the enhanced efficacy of TKIs against TCL may be attributable to mechanisms beyond targets like PDGFR and KIT. The finding that CPI-818, an inhibitor of the intracellular tyrosine kinase ITK, also achieved moderate efficacy (ORR = 0.67) further supports the notion that alternative signaling pathways are operative in cTCL (42). Nevertheless, these findings should be interpreted cautiously, as the evidence base remains limited by the small number of enrolled cTCL patients and the scarcity of available clinical studies.

Contemporary cancer chemotherapeutic protocols predominantly employ multi-agent regimens targeting complementary pathways—a strategy mirrored in current studies of cL treatment. Against this background, it should be noted that in the included clinical trials, Pan et al. (44) applied CCNU and TKIs (toceranib) combination therapy for cL, with an ORR reaching 0.67, suggesting that the combination has anti-tumor activity. While this demonstrates preliminary antitumor efficacy, validation through prospective randomized controlled trials remains imperative to establish clinical significance. Complementary *in vitro* experiments further elucidate TKIs combination strategies. Of five studies exploring the combination of TKIs with other drugs, two focused on combinations of cytotoxic agents. Zandvliet et al. (49) demonstrated that masitinib resensitized doxorubicin-resistant GL-40 cells (IC_50_ of doxorubicin on GL-40 cells: from 48ng/ml to 15ng/ml), suggesting TKIs-mediated reversal of chemoresistance in refractory cL. Mechanistic parallels emerge in the work of Chen et al. (51), where imatinib maintained potency against doxorubicin-resistant CLBL-8.0 cells (the resistance ratio in CLBL1–8.0 cells was 9.7 for doxorubicin compared with 0.9 for imatinib). Although these findings implicated TKIs as salvage therapies for treatment-resistant lymphoma, the underlying mechanisms, particularly potential inhibition of P-glycoprotein (P-gp)-mediated drug efflux, require systematic research through ABC transporter profiling and functional efflux assays (51). Of the five studies, two studies applied Ibrutinib (BTK inhibitors) and AS-605240 (PI3K inhibitors) to the CLBL-1 cell line, with results suggesting synergistic anti-lymphoma effects compared to single-agent treatments, indicating another promising strategy of using TKIs to overcome resistance (47). In addition to TKIs, there are inhibitors that target other signaling pathway components, such as PI3K inhibitors (56), JAK inhibitors (57), and NF-kappaB (NF-κB) inhibitors (58). Compared to normal canine B cells, genes involved in the PI3K/Akt/mTOR signaling pathway and the JAK-STAT pathway are significantly upregulated in cDLBCL, resembling the activated B-cell-like subtype of human DLBCL. In particular, the relationship between cell receptors in B-cell lymphoma and NF-κB deserves investigation. Bruton's tyrosine kinase (BTK) is primarily expressed in B cells but not in T cells or plasma cells (59). After activation by LYN (Src family tyrosine kinase) or spleen tyrosine kinase (SYK), BTK triggers a series of cellular physiological processes that lead to the activation of the NF-κB pathway. In this signaling pathway, two points can be highlighted: one is BTK itself, and the other is the downstream NF-κB effector. The former is one of the targets of the aforementioned TKIs, and NF-κB inhibitors that also inhibit this signaling pathway might be used in combination with BTK inhibitors for targeted therapy to enhance efficacy. Many BTK-targeted inhibitors have been or are being developed and have shown benefit in clinical trials for human B-cell lymphoma, particularly in patients with relapsed or refractory lymphoma. Perhaps this combined targeted therapy of TKIs and NF-κB inhibitors could provide a reference for the treatment of cL (60).

In the context of resistance, it should be noted that in the included clinical trials, Yamazaki et al. (43) used Toceranib monotherapy for MDR dogs, with an overall response rate (ORR) of 40% (2/5) for MDR lymphoma, highlighting the therapeutic potential of using TKIs for MDR lymphoma in dogs. Collectively, TKIs exhibit promising therapeutic value against chemotherapy-resistant cL through both single-agent and combination approaches. These findings not only validate the therapeutic potential of targeted agents in cL but also establish a conceptual framework for developing multidrug regimens that simultaneously block primary oncogenic drivers and compensatory resistance pathways, ultimately aiming to improve outcomes in treatment-resistant cases. Regarding resistance, it is widely believed that chemoresistance is a major barrier to successful cL management with traditional protocols, but little is known about its mechanisms. Consistent with human oncology, most research focuses on ABC transporters, particularly the P-gp efflux pump encoded by ABCB1, which is highly expressed in some lymphoma-affected dogs (61) and associated with drug-resistant phenotypes (62, 63). When dogs develop recurrent or acquire drug-resistant phenotypes, they express high levels of P-gp (63, 64). Dogs with high P-gp expression before chemotherapy correlate with reduced response rates and shorter overall survival (63). Additionally, the expression of P-gp observed in spontaneous cL paralleled those documented in human NHL (64), reinforcing the translational value of cL as a comparative model for human NHL studies.

In the included *in vitro* experiments, Zand Vliet et al. (50) revealed limited efficacy of TKIs against the GL-40 drug-resistance cell lines. The IC_50_ values were comparable to the GL-1 reference group (*P* > 0.05). The findings indicated that certain cell lines may possess intrinsic, target-independent resistance mechanisms. Suter et al. (52) found that Lestaurtinib, which targets FLT3, only had a significant anti-proliferative effect in *FLT3*-mutated GL-1 cell line, emphasizing the need for targeted drug therapy based on mutation status, and suggesting that mutation testing in dogs before TKIs treatment is an important indicator for the use of appropriate drugs.

While discussing the efficacy of TKIs in cL, we must also mention the potential therapeutic risks associated with TKIs. These risks mainly include the development of resistance and the occurrence of adverse events. Regarding resistance, there is extensive research in human medicine, and potential solutions have been explored (65). TKIs-targeted treatment resistance mechanisms primarily include acquired resistance and primary resistance, with acquired resistance mechanisms including target mutations, target gene amplification, loss of target gene mutations, activation of alternative signaling pathways (off-target), histological transformation (off-target), and changes in the tumor microenvironment (off-target). These findings underscore the need for strategic optimization of TKIs applications—both as monotherapy and in rational combinations—to maximize therapeutic efficacy and minimize adverse effects against cL. Current evidence supports that combinatorial therapies, including targeted drug synergism (e.g., rational polytherapy combining TKIs with NF-κB /PI3K-Akt pathway inhibitors), chemotherapy integration (e.g., CHOP-TKIs protocols to enhance cytotoxic effects), and immunotherapy combinations (e.g., immune checkpoint inhibitors with TKIs to restore antitumor immunity), are important strategies to overcome TKIs-targeted treatment resistance (65). Regarding adverse events, reported in human medicine, the frequency of TKIs-induced adverse reactions is increasing, with cardiac involvement being the most serious complication (66). There are few studies on TKIs treatment-related adverse reactions in cL, but prospective studies suggest that toceranib phosphate can disrupt the dog's hypothalamic-pituitary-thyroid axis (67). Clinical trial data of various TKIs for lymphoma further reveal toxic profiles in canine subjects: mild-to-moderate adverse reactions are predominantly gastrointestinal manifestations (anorexia, vomiting, diarrhea, weight loss), hematological suppression (neutropenia, thrombocytopenia, anemia, leukopenia) and mild liver function abnormalities (elevated ALP, ALT), while severe grade 3–4 toxicities mainly manifest as life-threatening gastrointestinal injury, severe myelosuppression and hepatotoxicity. Notably, the c-KIT/PDGFR inhibitor masitinib carries prominent high-grade gastrointestinal and hepatic toxicity in canine T-cell lymphoma models, and the combination of toceranib with CCNU markedly elevates the risk of severe hematological adverse events. All detail information has been shown in Table 5. Given the conservation of gene sequences between humans and dogs, with the widespread use of TKIs in the treatment of cL, rigorous veterinary surveillance of TKIs-associated adverse events (AEs) documented in human oncology is necessary.

**Table 5:** The side effects of different TKIs in clinical trials.

References	Diseases	Drug	Mild to moderate adverse reactions (Grade 1–2)	Severe adverse reactions (Grade 3–4)
Honigberg et al. (36)	B cell lymphoma	PCI−32765 (BTK inhibitors)	Not reported	Not reported
Gardner et al. (37)	B cell non–Hodgkin lymphoma	ACP−196 (BTK inhibitors)	Some anorexia	Not reported
Harrington et al. (38)	B cell non–Hodgkin lymphoma	Acalabrutinib (BTK inhibitors)	Vomiting (4/20), diarrhea (1/20), anorexia (5/20), lethargy (1/20), and slight body weight loss (1/20)	No drug–related grade 3–4 toxicity; 2 severe complications were confirmed unrelated to the study drug
Wang et al. (39)	B cell lymphoma	SY−1530 (BTK inhibitors)	Not reported	Not reported
Hermine et al. (40)	Peripheral T cell Lymphoma	Masitinib (c–KIT and PDGFR inhibitors)	Not reported	Not reported
Holtermann et al. (41)	Canine epitheliotropic T cell lymphoma	Masitinib (c–KIT and PDGFR inhibitors)	Anemia (3/10), neutropenia (1/10), slightly elevated ALP (1/10), thrombocytopenia (1/10), hypoalbuminemia (1/10), and slight body weight loss (1/10)	One case of grade 4 diarrhea, grade 3 vomiting and anorexia combined with hypoalbuminemia, resulting in euthanasia; grade 3 hepatic toxicity observed (1/10)
Thamm et al. (42)	T cell Lymphoma	CPI−818 (ITK inhibitors)	Hyporexia (1/3), vomiting (1/3), lethargy (1/3), polydipsia (1/3), biopsy site swelling (1/3), lymphopenia (1/3), hyperproteinemia (2/3), increased BUN (1/3), hypochloremia (1/3)	Not reported
Yamazaki et al. (43)	Multidrug–resistant (MDR)canine B and T cell lymphoma	Toceranib (Palladia) (PDGFR, VEGFR, and KIT inhibitors)	Diarrhea (1/5), mild neutropenia (1/5)	Not reported
Pan et al. (44)	13 tumor–bearing dogs (including lymphoma)	CCNU and toceranib	Neutropenia (1/13), lethargy (3/13), thrombocytopenia (4/13), diarrhea (2/13), elevated alt (6/13), hypertension (8/13), anorexia (4/13), vomit (2/13), nausea (2/13)	Neutropenia (8/13), thrombocytopenia (1/13), elevated ALT (4/13), hypertension (1/13), anorexia (1/13), muscle weakness (1/13)
Baba et al. (45)	Canine epitheliotropic lymphoma	Oclacitinib (JAK1, JAK2, JAK3, tyrosine kinase 2 inhibitors)	Mild transient leukopenia (1/8)	Not reported

This meta-analysis is not without its limitations. Firstly, the small number of clinical trials (*n* = 11) may restrict the generalizability of these findings across diverse canine populations. Secondly, since all included clinical trials were single-arm trials without comparison to standard treatment or other treatment methods, the relative effectiveness of TKIs treatment cannot be assessed, nor can the influence of natural recovery or placebo effects be excluded, affecting the evaluation of treatment efficacy. Thirdly, the single-arm clinical trials limit their ability to establish causal relationships between observed treatment and TKIs administration, as it precludes comparative analysis with control groups. Furthermore, the predominance of small, single-arm studies limits the robustness of current evidence and underscores the need for larger multicenter clinical trials to confirm the efficacy and safety of TKIs in canine lymphoma. The quality of the included trials was assessed using the MINORS scale, with variations in study quality (median score:13; range: 12–13). Although the heterogeneity analysis showed low heterogeneity (*I*^2^ = 33%) between studies and little heterogeneity in cBCL subgroup (*I*^2^ = 0) and cTCL subgroup (*I*^2^ = 0), there could still be heterogeneity due to factors such as disease stages, substages, combination regimes, prior treatments and immunophenotypes. Among these potential sources of heterogeneity investigated, immunophenotype emerged as the only factor showing a statistically significant difference in subgroup analysis (cBCL: 0.27 *vs*. cTCL: 0.63). Despite extensive subgroup analysis, residual heterogeneity may persist due to other factors such as subjective RECIST assessment, insufficient follow-up duration and detailed lymphoma subclassifications. However, future studies employing standardized protocols for cL patients, treatment administration, and outcome assessment will be essential to minimize inter-study variability and enhance the reliability of comparative analyses.

## 5 Conclusions

In conclusion, TKIs may exhibit moderate anti-lymphoma activity in dogs, with potentially greater benefit observed in canine TCL than in canine BCL. However, their clinical adoption requires rigorous validation through prospective randomized multicenter trials, biomarker integration, and combinatorial approaches to mitigate resistance and toxicity. Moreover, this study underscores cL's value as a translational model for human NHL, bridging species-specific therapeutic innovation.

## Data Availability

The original contributions presented in the study are included in the article/Supplementary Material, further inquiries can be directed to the corresponding authors.
