# Supplementary material for: Tyrosine kinase inhibitors and molecularly driven therapeutic approaches for canine lymphoma: a systematic review and meta-analysis
Source: Front Vet Sci. 2026 Aug 12;13:1852091. doi: 10.3389/fvets.2026.1852091 (PMC13506451; doi:10.3389/fvets.2026.1852091)
Supplement: Supplementary file 1 [file Supplementary_file_1.docx]

**Search strategy (PubMed as an example)：**

((("Dogs"[Mesh] OR ((Dog) OR (Canis)) OR ((((((Disease, Dog) OR (Diseases, Dog)) OR (Dog Disease)) OR (Canine Diseases)) OR (Disease, Canine)) OR (Diseases, Canine)) OR (Canine)) AND (("Lymphoma/drug therapy"[Mesh] OR "Lymphoma/therapy"[Mesh] OR "Lymphoma/veterinary"[Mesh]) OR (((((((((((((Lymphomas) OR (Germinoblastoma)) OR (Germinoblastomas)) OR (Lymphoma, Malignant)) OR (Lymphomas, Malignant)) OR (Malignant Lymphoma)) OR (Malignant Lymphomas)) OR (Reticulolymphosarcoma)) OR (Reticulolymphosarcomas)) OR (Sarcoma, Germinoblastic)) OR (Germinoblastic Sarcoma)) OR (Germinoblastic Sarcomas)) OR (Sarcomas, Germinoblastic)))) AND ((("Tyrosine Kinase Inhibitors/administration and dosage"[Mesh] OR "Tyrosine Kinase Inhibitors/pharmacology"[Mesh] OR "Tyrosine Kinase Inhibitors/therapeutic use"[Mesh]) OR (((((Inhibitors, Tyrosine Kinase) OR (Kinase Inhibitors, Tyrosine)) OR (Tyrosine Protein Kinase Inhibitors)) OR (Tyrosine Kinase Inhibitor)) OR (TKI Tyrosine Kinase Inhibitors))) OR (("Protein-Tyrosine Kinases/antagonists and inhibitors"[Mesh]) OR ((((((((((((((((((((((Kinases, Protein-Tyrosine) OR (Protein Tyrosine Kinases)) OR (Protein-Tyrosine Kinase)) OR (Kinase, Protein-Tyrosine)) OR (Protein Tyrosine Kinase)) OR (Tyrosine Kinase)) OR (Kinase, Tyrosine)) OR (Tyrosine-Specific Protein Kinase)) OR (Kinase, Tyrosine-Specific Protein)) OR (Protein Kinase, Tyrosine-Specific)) OR (Tyrosine Specific Protein Kinase)) OR (Tyrosylprotein Kinase)) OR (Kinase, Tyrosylprotein)) OR (Tyrosine Protein Kinases)) OR (Kinases, Tyrosine Protein)) OR (Protein Kinases, Tyrosine)) OR (Tyrosine Protein Kinase)) OR (Kinase, Tyrosine Protein)) OR (Tyrosine-Specific Protein Kinases)) OR (Kinases, Tyrosine-Specific Protein)) OR (Protein Kinases, Tyrosine-Specific)) OR (Tyrosine Specific Protein Kinases))))) NOT (("Mastocytoma"[Mesh]) OR ((((((Mastocytoma, Benign) OR (Benign Mastocytoma)) OR (Extracutaneous Mastocytoma)) OR (Extracutaneous Mastocytomas)) OR (Mastocytomas, Extracutaneous)) OR (Mastocytoma, Extracutaneous))) AND ("2004/01/01"[PDAT] : "2026/01/01"[PDAT])

**Figure S1** Forest plot showing the results of the meta-analysis of overall response rate (ORR) in cL. Studies that did not clarify whether patients received monotherapy or combination therapy were excluded from this subgroup analysis. The plotted proportion represents the ORR, defined as the number of patients achieving complete or partial response (CR+PR) divided by the total in each study (n). Pooled analysis shows a pooled ORR of 0.44 for monotherapy and 0.67 for combination therapy, *P*-value for subgroup difference greater than 0.05 under random-effects model. The overall heterogeneity across all included studies was moderate (*I²* = 33.0%), and subgroup heterogeneity for monotherapy was mild (*I²* = 37%). Squares and diamonds represent individual and pooled estimates, respectively, with 95% CI.

**Figure S2** Forest plot showing the results of the meta-analysis of overall response rate (ORR) in cL. Studies without clear records of multidrug resistance (MDR) status were excluded from this subgroup analysis. The plotted proportion represents the ORR, defined as the number of patients achieving complete or partial response (CR+PR) divided by the total in each study (n). Pooled analysis shows a pooled ORR of 0.45 for non-MDR subgroup and 0.40 for MDR subgroup, *P*-value for subgroup difference greater than 0.05 under random-effects model. Moderate overall heterogeneity (*I²* = 26.5%) was observed across all included studies, with mild heterogeneity within the non-MDR subgroup (*I²* = 34.5%). Squares and diamonds represent individual and pooled estimates, respectively, with 95% CI.

**Figure S3** Forest plot showing the results of the meta-analysis of overall response rate (ORR) in cL. Studies with unreported tumor stage information were excluded for this stratified analysis. The plotted proportion represents the ORR, defined as the number of patients achieving complete or partial response (CR+PR) divided by the total in each study (n). Pooled analysis revealed pooled ORRs of 0.36 (stage IIIa), 0.50 (stage Va), 0.00 (stage IIIb), 0.00 (stage IVb), and 0.25 (stage IVa); the *P*-value for subgroup difference was greater than 0.05 under random-effects model. No significant overall heterogeneity was detected across all studies (*I²* = 0.0%). Squares and diamonds represent individual and pooled estimates, respectively, with 95% CI.

**Figure S4** Forest plot showing the results of the meta-analysis of overall response rate (ORR) in cL. Cases without definitive pathological subtype classification were excluded from this subgroup meta-analysis. The plotted proportion represents the ORR, defined as the number of patients achieving complete or partial response (CR+PR) divided by the total in each study (n). Pooled ORRs varied across pathological subtypes, ranging from 0.27 (DLBCL_IB) to 0.66 (CTCL); the *P*-value for subgroup difference was less than 0.05 under random-effects model. Minimal overall heterogeneity (*I²* = 2.0%) was identified across all included studies, and no substantial heterogeneity was observed within each individual subtype subgroup. Squares and diamonds represent individual and pooled estimates, respectively, with 95% CI.

**Figure S5** Forest plot showing the results of the meta-analysis of overall response rate (ORR) in cL. Studies failing to report whether patients received prior anti-tumor therapy were excluded from this stratified analysis. The plotted proportion represents the ORR, defined as the number of patients achieving complete or partial response (CR+PR) divided by the total in each study (n). Pooled analysis shows a pooled ORR of 0.47 for treatment-naive patients and 0.34 for patients with prior treatment, *P*-value for subgroup difference greater than 0.05 under random-effects model. Mild overall heterogeneity (*I²* = 8.3%) was found across all studies, while substantial heterogeneity existed within the treatment-naive subgroup (*I²* = 67.6%). Squares and diamonds represent individual and pooled estimates, respectively, with 95% CI.
